# Cancer, metastasis, and the epigenome

**DOI:** 10.1186/s12943-024-02069-w

**Published:** 2024-08-02

**Authors:** Saurav Kiri, Tyrone Ryba

**Affiliations:** 1 College of Medicine, University of Central Florida, 6850 Lake Nona Blvd., Orlando, 32827 Florida USA; 2 Department of Natural Sciences, New College of Florida, 5800 Bay Shore Rd., Sarasota, 34243 Florida USA

**Keywords:** Metastasis, Epigenetic, Chromatin state, EMT, Topology, Transcription

## Abstract

Cancer is the second leading cause of death worldwide and disease burden is expected to increase globally throughout the next several decades, with the majority of cancer-related deaths occurring in metastatic disease. Cancers exhibit known hallmarks that endow them with increased survival and proliferative capacities, frequently as a result of de-stabilizing mutations. However, the genomic features that resolve metastatic clones from primary tumors are not yet well-characterized, as no mutational landscape has been identified as predictive of metastasis. Further, many cancers exhibit no known mutation signature. This suggests a larger role for non-mutational genome re-organization in promoting cancer evolution and dissemination. In this review, we highlight current critical needs for understanding cell state transitions and clonal selection advantages for metastatic cancer cells. We examine links between epigenetic states, genome structure, and misregulation of tumor suppressors and oncogenes, and discuss how recent technologies for understanding domain-scale regulation have been leveraged for a more complete picture of oncogenic and metastatic potential.

## Introduction

Despite advances in detection and treatment, cancer remains a major public health crisis across the globe. In the United States (USA), cancer has been the perennial second leading cause of death since 1937 [1]. Throughout 2020 and 2021, USA cancer deaths eclipsed those attributed to COVID-19 in spite of the ongoing worldwide pandemic, with roughly 600,000 deaths attributable to cancer in 2020 as compared to around 350,000 from COVID-19 [2, 3]. It was estimated that in 2024, 2 million new cancer cases would be diagnosed in the United States alone, along with over 600,000 estimated deaths [4]. Current extrapolations of incidence and mortality in the USA predict upwards of 2 million new cases per year diagnosed nationally by 2030 [5] and between 2.2 and about 3 million cases diagnosed per year by 2050 [6], with the rate of increase in incidence expected to outpace population growth during this time [5]. Males and females born in the USA today have roughly a 40% and 38% chance of being diagnosed with cancer in their lifetime, respectively [7].

Similar trends in cancer incidence exist on the international stage. In 2020, over 19 million cancer cases and almost 10 million cancer deaths were reported globally [8], increased from 14.1 million new cases and 8.2 million deaths from cancer in 2012 [9]. By 2070, it is expected that over 34 million cancer cases per year will be diagnosed; lower-income countries are projected to be disproportionately affected with an estimated 400% increase in cancer incidence in these countries within the next 50 years [10]. Among the cancer types expected to see the greatest increase in global incidence are breast, lung, and colorectal cancer (CRC). Models predict that the number of yearly CRC cases will reach 3.2 million by 2040 (as compared to 1.9 million in 2020) [11], and breast cancer incidence will reach similar levels by 2050 (up from 2.26 million in 2020) [8, 12]. Lung cancer, the current most prevalent type, will reach 3 million cases by 2035, with deaths expected to increase in all areas of the world [13]. The proposed patterns of cancer prevalence and mortality can be ascribed in part to general population growth, increased life expectancy, and associated risk factors including changes in diet and tobacco usage [11, 13, 14].

## Cancer epidemiology

### Disease burden

A more complete picture of cancer burden can be achieved by considering disability-adjusted life years (DALYs), where one DALY is defined by the World Health Organization as the equivalent of one year of life at full health lost. In 2019, cancer was calculated to cause 250 million DALYs, ranking second out of 22 in DALYs for diseases classified as Level 2 by the Global Burden of Disease project, only surpassed by cardiovascular disease (393 million DALYs) [15]. In that year, lung cancer alone accounted for 1.8% of all global DALYs and is among the top 10 contributors to increasing disease burden from 1990 to 2019—displaying a 69.1% absolute increase in DALYs over this period, despite a decrease in age-standardized rates during this time [16]. While age-standardized incidence and mortality have not increased globally from 2010 to 2019, the projected estimates for cancer described above and the upward trend in cancer burden in lower-income countries in the past decade call for continuing improvement in tools for screening and treatment [15].

### Clinical outcomes of metastatic cancer

Metastasis occurs when cancerous cells detach from a primary tumor and establish a new malignancy in a distinct tissue type, simultaneously adapting to the selective pressure imposed by the new microenvironment. It has been estimated that up to 90% of cancer deaths are due to metastatic cancer [17]. Cancer metastases frequently result in poor 5-year survival rates; retrospective studies demonstrated 5-year survival rates below 5% for hepatic metastasis [18] and about 5% and 8% survival for distant metastases from the lung in males and females, respectively [19]. While metastatic breast cancer survival has been improving, overall survival in hormone receptor (HR)/ERBB2(HER2)-negative metastatic breast cancer is estimated to be only 14 months [20]. Treatment and survival can be further impaired by compound metastasis. A retrospective study of the Danish Cancer Registry established that metastases to the bone co-occurring with other metastases produced a significantly higher risk for mortality in seven out of ten tested cancers, with 5-year survival rates following a diagnosis of bone metastasis below 10% for all primary cancer types except breast cancer [21]. The especially poor outcomes in metastatic cancer warrant a proportionally greater focus on the cellular processes underlying this critical event.

## Processes and features of carcinogenesis

Cancer constitutes a variety of diseases caused by acquired genome modifications leading to unchecked and inappropriate growth. While cancer itself is a substantially broad classification of cellular state, Hanahan and Weinberg’s identification of common features or “hallmarks” at the turn of the 21st century was foundational in organizing modern research and knowledge in oncology [22]. They initially described several key traits held by all cancers: independence from regulated growth signaling, the ability to spread to other tissues, directed vascularization, evasion of replicative limits, and downregulation of pathways promoting programmed cell death. Roughly a decade later, they expanded these key features to also include the anti-immune response of tumors, genome instability, and the misregulation of metabolism to also serve as prominent features of carcinogenesis [23]. Most recently, the role of epigenetic modification and phenotypic malleability have been emphasized as processes or drivers of cancer development [24]. Of these, constitutive proliferation, genome instability, and phenotypic malleability are highly relevant for metastatic cancer.

### Improper checkpoint and growth control

**Fig. 1:**
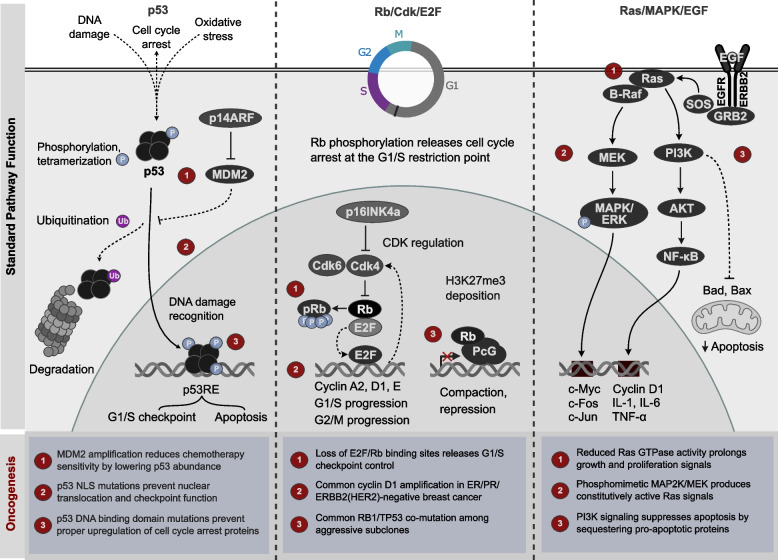
Oncogenic loss of checkpoint and growth control. (Left) A p53 tetramer mediates G1/S checkpoint and apoptotic responses to DNA damage and other stressors through transcription of p53 response element (p53RE) target genes. Binding of p14ARF (CDKN2A alternate reading frame) to MDM2 inhibits p53 ubiquitination and degradation. (Middle) Rb promotes G1/S progression through E2F transcription factors, transcribing cyclins and activating cylin-dependent kinases that in turn activate Rb. Rb/PcG complexes also repress transcription through H3K27me3 deposition from Polycomb group complexes (PcG). (Right) A phosphorylation cascade proceeds from Ras through either B-Raf, MEK, and MAPK/ERK proteins, or PI3K, AKT, and NF-$\kappa$B, activating growth, proliferation, and immune responses. (Bottom) Common alterations that disrupt checkpoint function, increase proliferative signals, or affect cell cycle regulation and growth through alterations in p53, Rb, or Ras signaling, promoting oncogenesis

The rapid replication of tumor cells is a fundamental feature of carcinogenesis. Evasion of the cell cycle control checkpoints that govern cell division is one of many mechanisms that promote this aggressive growth (Fig. 1). We thus begin by exploring the deleterious inactivation of tumor suppressors that undermine cell cycle control.

#### Mutation of TP53

Dysfunction of the tumor suppressor gene TP53, encoding the transcription factor p53, is estimated in at least 50% of all cancer cases, and contributes to cellular growth by preventing proper G1-S checkpoint control [25]. Cytoplasmic p53 is localized to the nucleus in response to DNA damage through interactions between its nuclear localization signal regions and the importin $\alpha$ and $\beta$ proteins; mutations in these regions of p53 and deletions of the nuclear localization signal-binding region of importin $\alpha$ lead to translocation of p53 to the cytoplasm, inhibiting its checkpoint function [26]. Mutations are most commonly found in the central DNA-binding domain of p53 [27] which disrupt its wild-type conformation and prevent proper upregulation of cell cycle arrest proteins. Further, several mutant forms of p53 are dominant over the wild-type counterpart, assembling in inactive mutant-wild type heterotetramers and activating growth-promoting genes such as telomerase reverse transcriptase (TERT) [28, 29].

Natively, p53 is found at low concentrations as it engages in a negative autoregulatory feedback loop by transactivating the ubiquitin protein ligase MDM2. MDM2 ubiquitinates C-terminal lysine residues of p53, triggering p53 degradation by the proteasome [30].

Counterintuitively then, amplification of MDM2 confers a fitness advantage to tumors against chemotherapy. High levels of MDM2 can constitutively keep wild-type p53 levels low, inhibiting the effect of chemotherapy agents designed to damage DNA and induce p53-mediated cell cycle arrest [31]. MDM2 can also contribute to consistent proliferation by independently acting as a positive regulator for the growth-promoting NF-$\kappa$B signaling pathway [32]. It is clear that a delicate balance of regulation is required for p53 to retain its anti-proliferative effects, and that undermining this balance via mutation or alterations in gene expression drastically increases the proliferative potential of a cell.

#### Inactivation of the retinoblastoma tumor suppressor

The Retinoblastoma (Rb)/E2F signaling axis is another tumor suppressing pathway abrogated in many cancer types (Fig. 1). The E2F family consists of several related transcription factors that promote progression through G1 and into S-phase, with a consensus binding site found in genes encoding cyclin A2, thymidine kinase, and DNA polymerase $\alpha$ [33]. In the absence of a mitogenic signal, Rb binds the E2F transactivation domain through its large pocket domain, masking its transcriptional control of cell cycle regulators [34].

Unlike p53, misregulation of Rb has direct epigenetic consequences, as Rb maintains long-term repression of E2F target genes through recruitment of chromatin remodeling enzymes. Rb can recruit Polycomb Group (PcG) proteins to E2F target promoters to deposit repressive histone 3, lysine 27 trimethylation (H3K27me3), thereby silencing gene expression [35]. Rb also recruits histone deacetylases (HDACs) to maintain E2F target promoters in a repressed state [35]. In this way, DNA-bound E2F serves a platform for Rb to recruit protein complexes to compact local chromatin and suppress transcription. In the presence of a positive growth signal, D-type cyclins are upregulated, leading to activation of cyclin-dependent kinases (Cdks) 4 and 6 to phosphorylate and inactivate Rb. Concomitantly, the E2F-responsive cyclin E is transactivated, leading to further phosphorylation and repression of Rb by cyclin E-associated Cdk2 [36].

Unsurprisingly, the Rb-E2F pathway is commandeered at several points in many tumors. Mutations in and upstream of RB1 have been well documented, with Rb frequently sustaining loss-of-function mutations that impair its ability to bind and regulate E2F [37]. Rb and TP53 mutations are also found in combination in aggressive cancer types prone to relapse, with the highest proportion of co-mutations (>75%) among small cell lung cancer cases with patient death [38].

In turn, some effects on Rb signaling are mediated through CDK activity. Over 20% of ER-positive/ERBB2(HER2)-normal and ERBB2-amplified exhibited cyclin D1 gene amplification in breast cancer patient data from The Cancer Genome Atlas (TCGA). Of all ER/PR/ERBB2 triple-negative breast cancer patients, 7.5% possessed whole-gene deletions of CDKN2A, encoding the p16INK4a competitive inhibitor of Cdk4 [39]. Loss-of-function mutations of CDKN2A can lead to overactive Cdk4 and increased suppression of Rb, in turn increasing E2F signaling. Just as with p53, the knockdown of a key tumor suppressor leads to accelerated passage through the cell cycle.

#### Pro-proliferative signaling: Ras/MAPK

In addition to the silencing of tumor suppressors, tumorigenic cell division is often accompanied by overactivity in growth-promoting pathways. One such vital pathway is the Ras/mitogen-activated protein kinase (MAPK) cascade (Fig. 1). The Ras protein family is populated by small GTP-binding signal transducers. Notably, the founding members NRAS, HRAS, and KRAS are known proto-oncogenes, with KRAS frequently mutated in pancreatic and lung cancers. Nearly 100% of advanced pancreatic ductal adenocarcinomas harbor a KRAS mutation, along with 25% of all screened tumors [40].

Ras/MAPK signaling proceeds through PI3K/AKT/mTOR or RAF/MEK/MAPK(ERK) pathways, each with members implicated in cancer type-specific misregulation and serving as corresponding anticancer therapy targets. Activation of the Ras/MAPK cascade begins with epidermal growth factor (EGF) receptor autophosphorylation. Phosphorylated tyrosine residues are bound by adapter protein Grb2, which interfaces with the guanine nucleotide exchange factor protein SOS. Ras prenylation promotes its localization to the plasma membrane, where it associates with SOS. In turn, SOS catalyzes the substitution of Ras-bound GDP for GTP and converts Ras to its active conformation [41]. This allows Ras to activate its effector proteins, including the Raf family of MAPK kinase kinases (MAP3Ks). At rest, Raf is autoinhibited through interactions between its N-terminal and C-terminal protein kinase domain, which is stabilized by the binding of regulatory 14-3-3 proteins [42]. However, activated Ras will bind to the Raf N-terminal Ras binding domain, which induces a conformational change in Raf that exposes a cysteine-rich domain containing phosphoserine inhibitory sites recognized by 14-3-3 proteins. De-phosphorylation and the exposure of the C-terminal kinase domain results in the activation of Raf [43]. Raf will then phosphorylate its MAPK kinase (MAP2K) target, MEK1, which will go on to phosphorylate the mitogen-activated kinase MAPK1/2 (ERK1/2). Unphosphorylated MAPK is sequestered in the cytoplasm via anchoring scaffold proteins, while activated MAPK can migrate to the nucleus and phosphorylate critical substrates, including the proto-onco transcription factors Jun (c-Jun), which includes cyclin D1 among many target genes, and c-Myc (MYC) [44].

Cascade signaling termination is catalyzed by protein phosphatases which will dephosphorylate active kinases and a Ras GTPase-activating protein (GAP). The intrinsic GTPase activity of Ras is significantly slow compared to the cellular timescale of Ras/MAPK signaling. Therefore, Ras requires the assistance of GAPs to hydrolyze GTP to GDP for deactivation [45].

Ras frequently suffers mutational insult in cancers, with mutation hotspots at residues 12, 13, and 61 [46]. Single amino acid substitutions of Gly12 or Gly13 sterically hinder the Ras GAP arginine residue from obtaining the proper orientation for hydrolysis, and mutation of the participating Gln61 residue similarly diminishes the GAP catalytic effect [45]. The net result of any of these three mutations is a constitutively active Ras that will continue to relay positive growth signal. B-Raf, among the most frequently mutated genes in human cancer [47], sustains gain-of-function through either point mutations that result in a constitutively active kinase domain, or through truncation of its N-terminal regulatory region, preventing autoinhibition [48]. These mutations could conceivably allow B-Raf to continue downstream pathway activation independent of Ras. Further downstream, various oncogenic mutations in MEK have also emerged: phosphomimetic mutation of Ser218 and Ser222 in the regulatory activation loop leaves the kinase domain persistently exposed and active, and a recent study additionally demonstrated the capacity for certain MEK mutations to induce autophosphorylation of the activation loop, achieving a similar result [49].

The downstream significance of the Ras/MAPK pathway has promoted development of numerous chemotherapy treatments against Ras, Raf, and MEK in an attempt to suppress this cascade in cancers. However, Ras remains a formidable challenge due in part to its ability to simultaneously activate the oncogenic phosphatidylinositol-3-kinase (PI3K)/protein kinase B (Akt) pathway, a central axis that supports proliferation through enhancing NF-$\kappa$B function and suppresses apoptosis by sequestering pro-apoptotic proteins (e.g., Bad and Bax) away from the mitochondria [50]. The dual activation of proliferative pathways by Ras (and increased survivability afforded by Akt signaling) contributes to drug resistance in cancers—research is currently ongoing to circumvent this problem [51].

#### The role of TERT in replicative immortality

Cancer cells thus have many tools to activate and maintain proliferative machinery. But is it possible for tumors to exploit this machinery in limitless capacity? In 1961, Hayflick and Moorhead discovered the bounded nature of cellular proliferation, demonstrating that human cells underwent cellular senescence after roughly 60 rounds of division (now termed the “Hayflick limit”)[52]. It was later discovered that a major contributor to cellular senescence is the incomplete replication of linear chromosomes due to the mechanism of DNA synthesis, coined the “end replication problem” by James Watson [53].

To catalyze elongation during DNA synthesis, DNA polymerases require an existing 3$^\prime$-hydroxyl group to coordinate an attack on the $\alpha$-phosphoryl group of an incoming deoxyribonucleotide. In eukaryotes, this is achieved through the DNA polymerase $\alpha$-primase complex, which catalyzes de novo addition of RNA-DNA primers that act as a scaffold for DNA polymerases $\epsilon$ and $\delta$ [54]. Normally, the RNA primers are degraded and replaced with deoxyribonucleotides. However, due to the nature of lagging strand synthesis, it is impossible to fill in cleaved RNA primers with the corresponding DNA sequence at the ends of chromosomes. Therefore, the net result is a shortening of the chromosomes after each round of replication. To prevent loss of protein-encoding regions during replication, tandemly repetitive non-coding DNA elements known as telomeres cap the ends of linear chromosomes. Telomeres themselves consist of a hexanucleotide repeat (5$^\prime$-$[TTAGGG]_n$-$3^\prime$) and associated shelterin proteins, which delineate telomeric DNA from damaged genomic DNA [55]. After repeated rounds of replication, telomeres become critically shortened such that the shelterin protein complex can no longer protect the free telomeric ends from activating DNA damage response proteins. At this point, cell cycle progression is arrested and senescence is activated [56].

However, senescence could theoretically be evaded upon the re-activation of the telomerase enzyme, which catalyzes the elongation of telomeres following their shortening. Indeed, TERT, the catalytic subunit of telomerase, is ubiquitously expressed across cancer types. TERT expression is suppressed in untransformed cells, but can become overactive in tumors due to mutation or epigenetic modification of its promoter. Cytosine-to-thymine transitions at -126 base pairs (bp) or -146 bp upstream of the translation start site creates a binding site for E26 transcription factors, which are hypothesized to activate the expression of TERT (as reviewed in [55]). Lee and colleagues also identified a region upstream of the TERT core promoter which was hypermethylated in cancer lines but unmethylated in normal lines lacking TERT expression, hypothesized to inhibit the binding of repressive proteins and allow for TERT transcription [57]. The expression of TERT endows cancer cells with replicative immortality, allowing oncogenic proliferation to persist indefinitely.

### Genomic instability

**Fig. 2:**
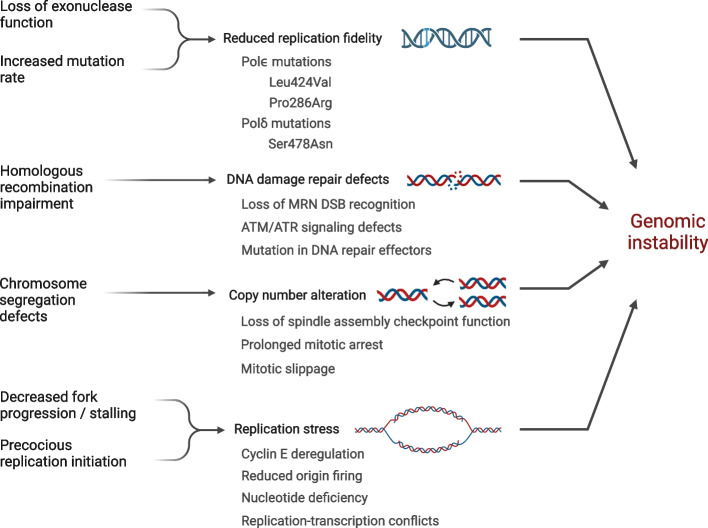
Origins of genomic instability. Defects in DNA damage recognition and repair, replication stress, copy number alteration, and reduced replication fidelity each contribute to genomic instability, which is self-perpetuating and exacerbated through rounds of cell division

While local coding mutations have well-established roles in destabilizing cell cycle regulation, broader genomic injury and epigenetic misregulation are significant drivers and results of oncogenesis. Genomic effects may manifest as replication stress, copy number alteration, defects in DNA damage repair, and exonuclease proofreading defects (Fig. 2).

#### Replication stress

A proposed mechanism for the emergence of genomic instability in tumors is oncogene-induced replication stress, broadly characterized by decreased progression and increased stalling of replication forks [58]. DNA replication begins with the licensing of replication origins through binding of the origin recognition complex to chromatin, which will subsequently recruit the key licensing factors Cdc6 and Cdt1. In turn, this machinery will recruit the replicative helicase MCM complex, forming the pre-replication complex (pre-RC). At the transition to S-phase, the MCM complex is phosphorylated by Cdks as well as the cell-cycle dependent kinase Cdc7-Dbf4, which allows for the binding of Cdc45 and the GINS protein complex to MCM, forming the DNA helicase holoenzyme (CMG); formation of the CMG is necessary to activate the ATPase and helicase activities of MCM in vitro [59]. DNA helicase activation leads to unwinding of duplex DNA and replication origin firing. To ensure that the genome is singly duplicated with high fidelity, formation of the pre-RC is under tight regulatory control of the cell cycle. Normally, Cdks, which are active in late G1 through M phase, are key inhibitory factors of pre-RC assembly. Phosphorylation of Cdc6 by Cdks triggers nuclear export, and Cdt1 is inhibited by direct binding with the geminin protein, whose regulation also falls under the jurisdiction of the anaphase-promoting complex/cyclosome (APC/C) [60]. Overall, this ensures that replication complexes do not re-form and fire after completion of replication in S-phase.

However, tumorigenic dysregulation of the cell cycle compromises this control. Deregulation of the oncogene cyclin E has been found to impair pre-RC assembly by reducing chromatin loading of the MCM complex, likely due to inhibition of the origin complex and/or its associated replication factors. Cyclin E overexpression reduced nuclear staining for the proliferating cell nuclear antigen (PCNA) sliding clamp, indicating fewer origins fired in S-phase [61]. Decreased MCM assembly and origin firing leaves cells hypersensitive to genomic instability, possibly by reducing the number of dormant origins that normally exist as a failsafe to rescue stalled replication [62]. Cyclin E may also contribute to genomic instability by forcing proliferation through increased E2F signaling without activating nucleotide biosynthetic pathways, though concomitant activation of nucleotide metabolism through Myc can rescue nucleotide depletion [63]. The early activation of replication that occurs with oncogenic signaling may also clash with transcriptional programs, leading to collisions between replication and transcription machinery. The torsional stress accumulated through head-on collision of a transcription bubble and replication fork can result in fork stalling and either collapse or reversal, which promote genomic instability through the creation of double-stranded breaks in the former case and through opportunities for recombination in the latter [64]. In many ways then, the fidelity of the replicative process can become compromised in cancer, thereby promoting genome fragility and fueling tumor progression.

#### ATM-mediated DNA damage response

An additional mechanism for genomic instability in cancers is mutational inactivation of the DNA damage response system responsible for rectifying genetic aberrations. Ataxia-telangiectasia mutated (ATM) recognition of double-stranded breaks (DSBs) is a frequent target of oncogenic mutations that impede homologous recombination (HR), resulting in error-prone non-homologous end-joining (NHEJ).

The main upstream kinases involved in DSB/single-stranded DNA (ssDNA) detection and signal transduction are the ataxia-telangiectasia mutated (ATM), ATM- and RAD3-related (ATR), and DNA-dependent protein kinase genes. The ATM-directed DNA damage response is initiated through binding of the MRE11-RAD50-NBS1 (MRN) complex to a DSB. At rest, ATM is assembled and autoinhibited as a dimer [65]. Upon binding activated NBS1 (NBN), ATM undergoes autophosphorylation to release the kinase domain, activating ATM through monomerization [66]. Activated ATM then phosphorylates histone H2A variant H2AX ($\gamma$H2AX) in nucleosome cores that flank the DSB, which is recognized by the BRCT motif of the mediator of DNA damage checkpoint protein 1 (MDC1). $\gamma$H2AX-bound MDC1 subsequently allows for the localization of E3-ubiquitin ligases RNF8 and RNF168 proximal to the DSB, catalyzing histone ubiquitination at the DSB and ultimately recruiting the breast cancer gene 1 (BRCA1) and p53 binding protein 1 (TP53BP1/53BP1) proteins [66, 67]. Prior to S-phase, TP53BP1 binds DSBs and promotes non-homologous end joining by blocking nucleolytic processing. After the G1-S transition, the effector protein CtIP (RBBP8) is phosphorylated and activated by Cdks, which will displace TP53BP1 in favor of BRCA1. This triggers resection of the DSB and initiates homologous recombination repair [68].

A critical role of ATM signaling is to halt cell cycle advancement in the presence of a DSB. ATM phosphorylates and activates p53 as well as checkpoint kinase 2 (CHEK2/CHK2), which will inactivate CDC phosphatases that activate Cdks, promoting cell cycle arrest and apoptotic cell death in extreme cases of damage [69]. Mutations in ATM increase breast cancer susceptibility. A meta-review by Stucci et al. reported a two- to thirteen-fold increase in breast cancer risk for individuals under 50 heterozygous for ATM mutation, as compared to wild-type homozygotes. Likewise, mutations in BRCA1 and BRCA2 have long been linked to breast cancer susceptibility, and germline mutations in the BRCA genes predispose to hereditary breast cancers [70]. Loss-of-function mutations in components of the ATM-mediated DNA damage response such as ATM, MRE11, and H2AX in tandem with overexpression of phosphatase Wip1, a negative regulator of this pathway, contribute to chemoresistance to crosslinking agents in head and neck carcinomas [71]. The misregulation of this response pathway may confer a selective advantage in tumorigenesis by allowing DSBs to induce chromosomal rearrangement (i.e., instability) and stochastically drive carcinogenesis. Yet this topic remains complex as targeted inhibition of the ATM pathway has yielded positive effects via chemotherapy sensitization or synthetic lethality in certain cancers, illustrating the double-edged nature of DNA repair in tumor progression [69].

#### Copy number alterations, aneuploidy and chromosomal instability

Copy number alterations have long been an identified feature of several cancers; prominent examples include ERBB2 (HER2) amplification, observed in nearly 20% of breast cancer cases [72], and MYCN amplification, estimated in 25% of neuroblastoma cases [73]. Karyotypic abnormalities are frequently identified in leukemias and lymphomas, such as trisomy 12 in chronic lymphocytic leukemia [74] and hyperdiploidy in B-cell acute lymphoblastic leukemia [75]. Such genetic anomalies can both result from and fuel genomic instability. In this section, we take a brief look at the relationship between alterations in copy number, genome stability, and carcinogenesis, focusing on aneuploidy.

Aneuploidy has the potential to confer tissue-specific fitness advantages that drive tumorigenesis based on the pattern of gain or loss by altering copy numbers of oncogenes and tumor suppressors, as well as creating opportunities for mitotic error. A prominent example is in trisomy 21 (Down syndrome). Patients with trisomy 21 have significantly increased risk for myeloid leukemias, but profoundly decreased risk of solid tumors [76]. Numerous molecular mechanisms have been proposed to explain this phenomenon. The hematopoiesis-stimulating factor RUNX1, located on chromosome 21, has recently been implicated in myeloid leukemia-associated Down syndrome. It was discovered that the presence of a third copy of chromosome 21 may alter the normal isoform ratio to favor RUNX1A, which in conjunction with mutant GATA1, can arrest differentiation of megakaryocytes and promote blast proliferation via MYC signaling [77]. A study with mice models by Ng and colleagues also demonstrated a role for trisomic overexpression of ERG, a transcription factor that participates in megakaryocytosis, in aberrant myeloproliferation [78]. While the anti-solid tumor effects of trisomy 21 have not yet been fully elucidated, proposed mechanisms include over-expression of RCAN1 and DYRK1A, which have been shown to inhibit VEGF-mediated angiogenesis [79].

Aneuploidy arises through errors in chromosomal segregation during mitosis, which can occur through dysfunction of the spindle assembly checkpoint (SAC; reviewed in [80]). This checkpoint normally serves to ensure that chromosomes are segregated faithfully and equally during anaphase of mitosis by checking that each kinetochore is occupied by a spindle microtubule before segregation occurs. The SAC chiefly serves to inhibit CDC20, an activator of the APC/C E3 ubiquitin ligase; the CDC20-bound APC/C will ubiquitinate mitotic regulators including cyclin B and securin, the latter of which normally sequesters separase. The release of separase leads to proteolysis of cohesin, allowing sister chromatids to separate [81, 82]. However, in the presence of a vacant kinetochore or unstable microtubule-kinetochore attachment, the mitotic checkpoint complex is assembled which inhibits CDC20 and subsequent APC/C activation [83].

Abuse or bypass of this signaling complex allows for constitutive proliferation and genomic instability. CDC20 has been implicated in cutaneous squamous cell carcinoma development through interactions with the Wnt/$\beta$-catenin pathway (discussed in Wnt-driven dedifferentiation section) [84], and inherited polymorphisms in the CDC20 gene have been identified in cases of familial malignant ovarian germ cell tumor [85]. Single-allele knockout at the MAD1L1 (MAD1) and MAD2L1 (MAD2) loci, participants in the SAC, have demonstrated synergistic effects in promoting tumorigenesis in mice models [86], and separate studies in mice have revealed that MAD2 mutants with a weakened mitotic checkpoint show greater load of chromosomal instability with embryonic lethality in double knockout mice [87]. However, it appears that a baseline level of checkpoint fidelity is required for cancer cell survival, as complete silencing of the SAC is lethal to tumor cells [88], again demonstrating the delicate balance of genome instability and carcinogenesis, as well as the role that impaired replicative fidelity may have on generating chromosomal instability.

Chromosomal mis-segregation has also been linked directly to DNA damage and structural anomalies. Janssen and colleagues discovered that lagging chromosomes are subject to structural damage following cytokinesis as evidenced by activation of the ATM damage response cascade, ultimately manifesting as double-stranded breaks or unbalanced rearrangements between chromosomes [89]. Impairment of the SAC or prolonged mitotic arrest by the SAC can, in some cases, lead to mitotic slippage, characterized by re-entry into interphase without cytokinesis. This process results in tetraploidization of the cell, which has been linked to chromosomal instability and cancer in p53-depleted lineages [90, 91] Aneuploidy itself has also been suggested to precipitate chromosomal instability by way of replication stress, possibly by creating increased demand for DNA replication factors during S phase [92]. Beyond these tumorigenic capabilities, quantitative abnormalities also create opportunities for chemotherapy resistance by altering gene expression programs of drug resistance pathways, including drug efflux pumps [93].

Dysfunction of the SAC is not the only mitotic source of genomic instability. Defects in cohesin, the protein responsible for sister chromatid attachment during mitosis, have been linked to aberrant chromosomal segregation during anaphase [94]; centrosome amplification events have been identified to compromise cellular polarity and promote mis-segregation through multipolar mitosis [95]; rapid telomere shortening can expose free ends of chromosomes, allowing for breakage-fusion-bridge cycles which may continuously generate instability. This process is characterized by fusion of sister chromatids to form dicentric chromosomes which create DNA bridges during anaphase [96]. Tension exerted on the fused chromosome at each centromere can result in asymmetric breakage, creating progeny with quantitative (e.g., amplifications, deletions) or structural (e.g., inversions) defects. The resultant damaged chromosomes may initiate another round of breakage, fusion, and bridging, propagating instability [96]. Although many tumors protect against telomere crisis via transactivation of TERT as previously discussed, telomere shortening and fusion events have been seen in clonal tumor populations with TERT activity [97], suggesting breakage-fusion-bridge cycling is possible in such cancers.

#### Compromised fidelity of replication and the mutator phenotype hypothesis

Even if the DNA damage response system is left intact, genetic lesions can still accrue when introduced with increased frequency. Mutations that amplify error rates in replicative DNA polymerases contribute to increased mutational burden. DNA polymerases can replicate parental DNA with high fidelity due in large part to their intrinsic $3^{\prime } \rightarrow 5^{\prime }$ exonuclease activity. This exonuclease activity is activated in response to nascent-parental DNA melting upon the incorporation of a non-Watson-Crick base pair, which flips the nascent strand into the exonuclease site for processing [98].

Loss of exonuclease activity is characteristic of certain hereditary cancers. Inheritance of the Leu424Val mutation in POLE, the catalytic subunit of polymerase $\epsilon$, or the Ser478Asn mutation in POLD1, the catalytic subunit of polymerase $\delta$, were found to increase risk for colorectal carcinoma [99]. Analysis of CRC tumor data from The Cancer Genome Atlas discovered that hypermutated CRCs harbored POLE lesions more than non-hypermutated counterparts [100]. More recently identified mutations in catalytic polymerase subunits impair replication fidelity without necessarily altering exonuclease catalytic capability. An important mutational hotspot in POLE identified in colorectal and endometrial cancers is Pro286Arg [101]. A recent study on the homologous mutation in yeast polymerase $\epsilon$ (Pro301Arg) found that this mutation conferred increased replicative efficiency by impairing elongation-exonuclease switching [102].

The observations that tumors display a broad spectrum of mutations in DNA damage response systems, checkpoint proteins, and replicative polymerases, coupled with findings that tumors harbor a significantly greater number of mutations than their non-transformed counterparts, led to the development of the mutator phenotype hypothesis almost 50 years ago by Lawrence Loeb [103]. Loeb suggested that the suppression of proteins that monitor genomic integrity occurs early in carcinogenesis, leading to generational acquisition of mutations and instability. At least some of these mutations would conceivably confer a selective advantage to a particular clone, leading to proliferation and the onset of tumor progression.

The examples of replication stress, chromosomal instability, and acquired deficiencies in replication represent genomic alteration both as an initiator of cancer development and as a consequence of persistent oncogenic processes. Although the web of genomic instability and its roles in cancer are still being untangled, emergent therapeutic insights hold promise in weaponizing genotoxic stress against cancer, and new understanding of chromatin structure, epigenetic drivers and therapies, and targeted epigenome screening have made significant recent progress [104]. CFI-402257 (Treadwell Therapeutics) is a recently developed inhibitor of the TTK serine/threonine kinase (also known as MPS1), a member of the previously discussed SAC, that has shown promise in suppressing spindle checkpoint activity and subsequently cancerous proliferation as part of combination therapy in a variety of tumors, including patient-derived cultures of hepatocellular carcinoma [105] and serous ovarian carcinoma [106]. Currently, a phase II clinical trial is underway for the treatment of ER+/ERBB2(HER2)- breast cancer with CFI-402257 and the estrogen receptor-degrading agent Fulvestrant (NCT05251714). Induction of genomic instability to facilitate cancer cell death is also the strategy employed by AZD1390 (AstraZeneca), a small molecule inhibitor of ATM. Early studies with mice proved AZD1390 to be effective in promoting radiosensitivity of glioblastoma multiforme by knocking down the double-stranded break repair pathway [107]. A phase I clinical trial is currently active to assess combination AZD1390 and radiation to treat malignancy of the brain (NCT03423628). PARP inhibitors have been well-established as bona fide suppressors of tumor growth due to their ability to limit the DNA damage repair capabilities of PARP1 and PARP2. The PARP enzymes play an especially vital role in stabilizing single-stranded breaks, stalled replication forks, and base excision repair [108–110]. Knockdown of PARP activity can overwhelm cancer cells with genotoxic stress and result in cell death. Many PARP inhibitors have already been approved (olaparib, niraparib, talazoparib) and are particularly effective against BRCA-mutant breast and ovarian tumors, possibly due to synthetic lethality of impaired DNA damage response pathways [111, 112]; in addition, olaparib is now considered first-line in maintenance therapy of pancreatic adenocarcinoma with a germline BRCA mutation following 16 weeks of progression-free survival with standard chemotherapy [113]. As our understanding of the mechanisms governing genomic instability in cancer deepens, so shall the arsenal of therapeutic targets to combat tumorigenesis.

### Phenotypic malleability

Cellular differentiation is the process in which precursor cells determined to a particular lineage adopt the specialized phenotype of that lineage. In the course of terminal differentiation, cells will exit the cell cycle, counter to the proliferative demand of cancer. The oncogenic escape from differentiation has been identified as a cancer hallmark and a potential advantage for disseminating tumor cells [24]. We explore Wnt and SOX2 signaling as two examples of how differentiation state affects tumorigenesis and prognosis.

#### Wnt-driven dedifferentiation

An important regulator of cell differentiation often hijacked in cancer is the powerful and highly conserved Wnt signaling pathway group. The Wnt family of proteins are palmitoylated glycoproteins that engage in signaling through the Frizzled receptor in an autocrine or paracrine fashion [114]. Wnt signaling plays a critical role in development of the anterior-posterior axis and cell fate specification as well as cellular proliferation, with c-Myc and cyclin D1 among downstream targets [115]. Wnt signaling consists of the $\beta$-catenin-dependent (canonical) and $\beta$-catenin-independent (noncanonical) pathways; here, we focus on the well-studied canonical pathway in oncogenesis.

**Fig. 3:**
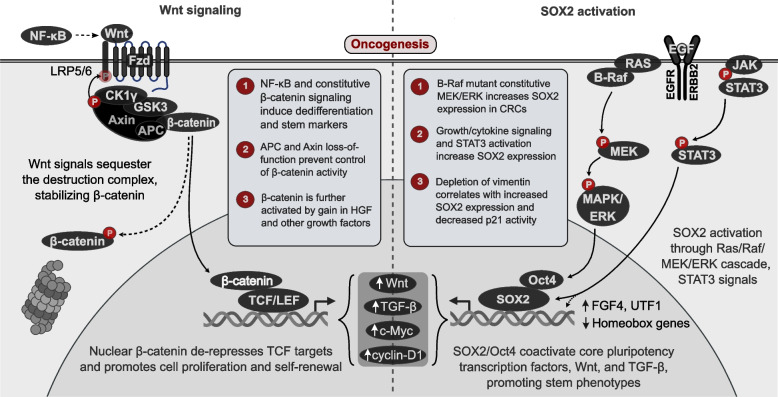
Phenotypic malleability and dedifferentiation. (Left) Wnt binding to Frizzled and LRP5/6 receptors triggers phosphorylation of LRP5/6 by GSK3 and CK1, which together with the recruitment of Dishevelled to Frizzled, binds Axin to the plasma membrane. This also sequesters the destruction complex to the dimerized receptors, stabilizing $\beta$-catenin and allowing its translocation to the nucleus to promote cell proliferation. (Right) SOX2 can be activated through various sources, including the Ras/Raf pathway or more directly though STAT3. SOX2/Oct4 complexes promote stem phenotypes and dedifferentiation by activating core pluripotency transcription factors

Wnt/$\beta$-catenin signaling begins with binding of Wnt to Frizzled (Fig. 3). The low-density lipoprotein receptor-related proteins LRP5/6 are also necessary co-receptors for $\beta$-catenin activation. Wnt binding to LRP5/6 induces phosphorylation of the intracellular domain by glycogen synthase kinase 3 (GSK3) and casein kinase 1$\gamma$ (CK1$\gamma$), which creates a binding site for Axin proteins and stabilizes $\beta$-catenin [116, 117]. Wnt-bound Frizzled can bind Dishevelled, which further recruits Axin to the plasma membrane [118]. In the absence of Wnt, $\beta$-catenin is normally localized to the cytoplasm and bound to a destruction complex, consisting of GSK3, casein kinase 1$\alpha$ (CK1$\alpha$), adenomatous polyposis coli (APC), protein phosphatase 2A (PP2A), and Axin. The phosphorylation of $\beta$-catenin by GSK3 and CK1$\alpha$ targets $\beta$-catenin for degradation via the E3 ubiquitin ligase $\beta$-TrCP [114]. However, binding of Wnt to its receptors disrupts the cytoplasmic destruction complex and allows $\beta$-catenin to enter the nucleus.

Upon entry to the nucleus, $\beta$-catenin will bind and activate T cell factor/lymphoid enhancer factor (TCF/LEF) transcription factors. Without $\beta$-catenin, TCF/LEFs serve as transcriptional repressors bound to Groucho repressor proteins that recruit chromatin compacting enzymes. However, $\beta$-catenin may also have context-dependent functions in human stem cell maintenance; in particular, loss of $\beta$-catenin or TCF4 may compromise intestinal stem cell production and renewal (as reviewed in [119]).

For this reason, Wnt is frequently leveraged in colorectal carcinomas. Schwitalla et al. found that in vivo constitutive activation of $\beta$-catenin through NF-$\kappa$B signaling induces expansion of stem cell-producing intestinal glands and increased binding of $\beta$-catenin to promoters of intestinal stem cell markers such as LGR5 and SOX9 in differentiated epithelial cells, indicating dedifferentiation. They also demonstrated the tumorigenic capacity of these dedifferentiated cells in organoid systems [120]. The tumor microenvironment likewise plays a key role in maintaining poorly differentiated, aggressive cancers. Secretion of hepatocyte growth factor by stromal cells leads to activation of hepatocyte growth factor receptor tyrosine kinase in colorectal carcinoma cells, which has been found to activate $\beta$-catenin and promote its translocation to the nucleus, augmenting aggressiveness [117, 121]. Loss-of-function mutation in SMAD4, a core mediator of the early tumor suppressive TGF-$\beta$ and bone morphogenetic protein (BMP) pathways, in tandem with increased $\beta$-catenin concentration, is sufficient for dedifferentiating intestinal absorptive cells to re-enter the cell cycle and promote neoplastic proliferation [122]. Critically, nonsense mutations in the APC gene upstream of the Axin binding site are considered initiating events in colorectal carcinoma, and it is estimated that over 80% of all CRC cases harbor such a mutation [123]. Truncated APC has attenuated ability to regulate $\beta$-catenin, allowing for possibly unchecked Wnt signaling. Taken together, these results clearly indicate that blocking of differentiation and proliferative signaling endowed by Wnt is indispensable for colorectal tumorigenesis and cancer progression.

#### Contribution of SOX2 to cancer cell stemness

The SRY-related HMG box (SOX) proteins are a family of conserved and developmentally vital transcription factors that share sequence homology with the founder protein, sex-determining region Y (SRY). In total, 20 distinct SOX genes have been identified in humans, divided into subfamilies A-H based on HMG DNA-binding domain homology. SOX gene subgroups show divergent functions in cell fate specification, maintenance of pluripotency, and somatic cell line development [124]. It is thus not surprising that SOX dysregulation contributes to cancerous transformation through numerous mechanisms (Fig. 3).

SOX2 is a master regulator of stem cell pluripotency along with its co-factor Oct4 (POU5F1). Indeed, SOX2 is one of the four Yamanaka factors (SOX2, Oct4, Klf4, and c-Myc), whose ectopic expression is sufficient to dedifferentiate mature mouse fibroblasts into induced pluripotent stem cells [125]. Evidence for the role of SOX2 in stem cell potential is found in SOX2 knockdown studies in vivo: disruption of SOX2 expression in the mouse embryo leads to differentiation of the inner cell mass (the source of embryonic stem cells) of the blastocyst to the trophectoderm phenotype, which will form the placenta [126]. SOX2 exerts its effects by forming a heterodimeric transcriptional regulation complex with the Oct4 transcription factor. SOX2 and Oct4 will bind their juxtaposed recognition motifs cooperatively through their HMG and POU domains, respectively [127]. The SOX2-Oct4 complex regulates pluripotency in part through control over downstream developmental transcription factors. Activating SOX2-Oct4 co-occupancy has been identified at the promoters of Wnt and TGF-$\beta$ pathway members, while repressive occupancy has been identified at several of the homeobox genes, regulators of cell specification [128].

SOX2’s capacity to regulate self-renewal and its cross-talk with other signaling pathways is co-opted in numerous cancer types. Depletion of SOX2 within melanoma cell populations expressing the cancer stem cell marker aldehyde dehydrogenase 1 limits their tumorigenic capacity and clonogenicity [129]. An in vitro study with the U87 glioma cell line demonstrated that transfection of glioma cells with the miRNA miR-378 led to increased expression of SOX2, the stem cell surface marker CD133, and induction of stem cell properties. The increase in SOX2 was attributed to miRNA-induced depletion of vimentin, an intermediate filament that positively regulates the cell cycle arrest and differentiation factor p21 in neuroblastoma [130, 131]. SOX2 expression also correlates with poor prognosis and tumor differentiation status in CRC. In vitro, CRC cells harboring Val600Glu-mutated B-Raf (which results in constitutive MAPK/ERK signaling) possess enhanced expression of SOX2, indicating possible crosstalk between the MAPK cascade and SOX signaling [132]. A study of breast cancer in mice also established cross-talk between the signal transducer and activator of transcription 3 (STAT3) pathway and SOX2 expression. STAT3, among other STAT transcription factors, is activated in response to growth or cytokine signaling. Yang et al. found that tumor-associated macrophages secreted epidermal growth factor (EGF) into the breast cancer tumor microenvironment, activating the EGF receptor tyrosine kinase (EGFR) in a paracrine fashion [133]. STAT3 binds the activated, autophosphorylated EGFR through its Src homology 2 domain, upon which it is also phoshorylated at Tyr705 by EGFR. This allows for homodimerization and transport into the nucleus, where it can exert its regulatory effects [134]. Yang et al. demonstrated this scheme ultimately leads to increased SOX2 expression and maintenance of the stem cell phenotype [133].

Critically, SOX2 lacks any small molecule binding domains, rendering it classically “undruggable”. Thus, SOX2 remains a dangerous transcription factor in cancer initiation and progression due to its role in maintaining aggressive, self-renewing cancer stem cells while simultaneously interacting with other oncogenic pathways.

## Epigenetic marks and genome architecture

Within the nucleus, DNA is found in complex with several proteins to form chromatin, and folds intricately to fit within the confines of the nuclear envelope. Consequently, topological and chemical properties of chromatin are important factors for gene expression. Key concepts in epigenetics and genome topology relate misregulation of transcription and chromatin state to carcinogenesis and the development of metastatic clones.

The importance of epigenetic state in carcinogenesis has been underscored by the prevalence of epigenetic lesions in cancer genomics studies [135] and the recognition of epigenetic reprogramming as a cancer hallmark [24]. With the development of targeted epigenetic therapies, reversible treatments of epigenetic states that drive misexpression, dedifferentiation and metastasis are in active development [136]. Here, we focus on markers of local chromatin state, transcriptional accessibility, and large-scale structural regulation.

### Histone modifications and chromatin state

#### The histone octamer

**Fig. 4:**
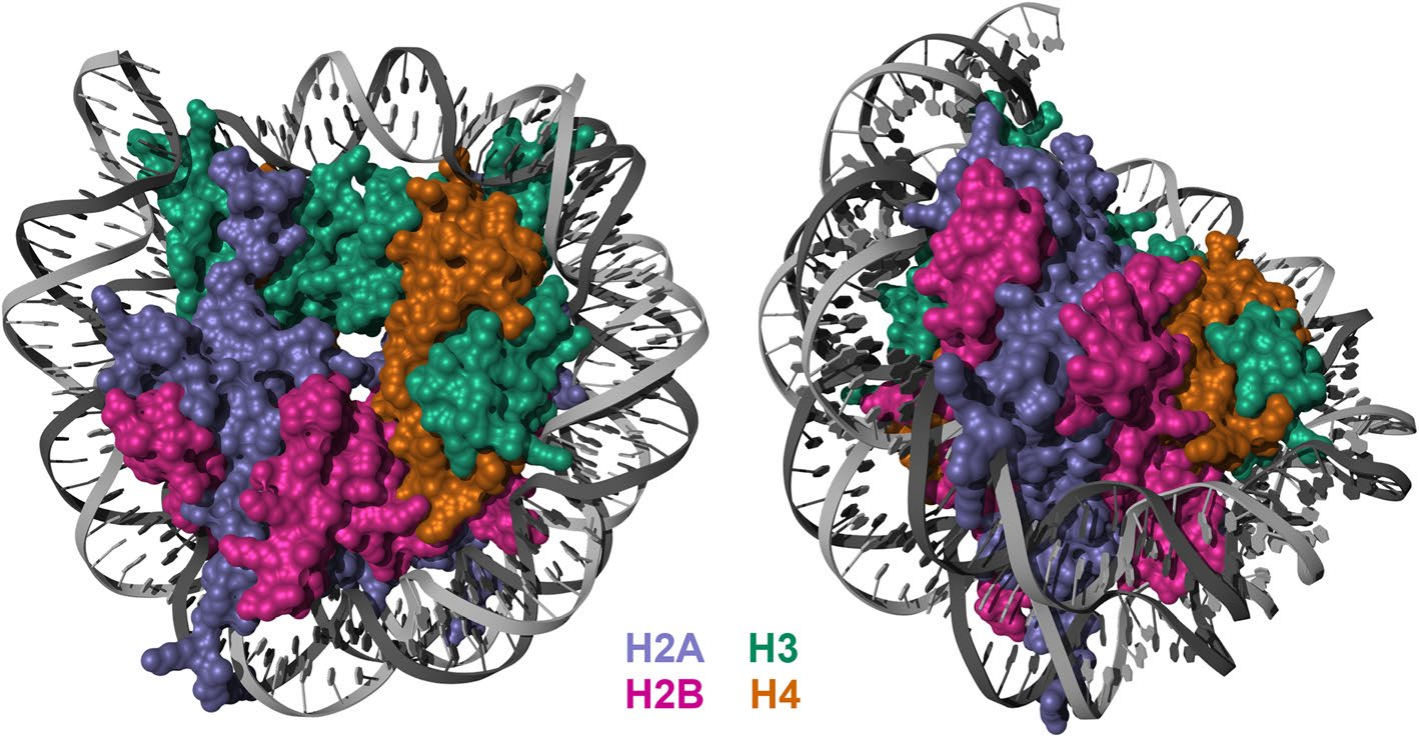
The nucleosome, consisting of about 146 bp of DNA wrapped around the histone octet. Histone tails protruding from the core particle serve as sites for covalent modification that alters gene expression. Structure image produced using PDB (ID: 7VZ4)

The fundamental unit of chromatin is the nucleosome, which consists of approximately 146 bp of DNA wrapped around a core of eight histone proteins (Fig. 4). The histones compromising the nucleosome core are H2A, H2B, H3, and H4. Additional histones (H1 or H5) take on the role of binding the linker DNA that chains nucleosomes together, thereby contributing to the condensation of chromatin into higher-order structure [137].

As wrapping DNA around histones naturally occludes the sequence held within that portion of the duplex, nucleosome structure and histone-DNA interactions serve as a platform for regulation by determining transcriptional accessibility. Post-translational modification of protruding histone tails plays a vital part in this regulatory process. Histone modification can affect transcription by either directly modifying electrostatic histone-DNA interactions, or creating or removing binding sites for transcriptional or chromatin state effectors [138]. We next explore prominent examples of each type of regulation, with particular attention to histone acetylation and enhancer activity and the regulation of chromatin states.

#### Histone methylation

Histone methylation occurs on the $\epsilon$-amino group of lysine or on the guanidinium group of the arginine side chain. Lysine may be mono-, di-, or trimethylated, whereas arginine can undergo mono- or dimethylation. Methyl group addition mediates binding sites for chromatin-interacting proteins [138]. In regulating transcription, histone methylation can be activating or repressing depending on the histone and residue. H3K27me3 is associated with repression through binding at promoters, throughout the gene body, and across broader repressive domains. H3K27me3 is established by and maintains suppression of these regions through Polycomb Repressive Complex (PRC) 1 and 2, with PRC2 acting as the catalytic complex for H3K27me3 deposition. The PRC2 core complex consists of the EZH2 histone methyltransferase (HMT), EED, and SUZ12 [139]. The HMT activity of EZH2 is endowed by its SUV39 SET domain, while the WD40 tandem repeats of EED form a cage-like structure that allows it to bind to repressive methyllysine marks and potentially allosterically activate EZH2, promoting H3K27 methylation at neighboring nucleosomes [140]. PRC2-catalyzed H3K27me3 is then recognized by the chromobox (CBX) subunit of PRC1, resulting in targeted monoubiquitination of H2A Lys119 by the RING finger E3 ubiquitin ligase subunits RING1A/B (RING1/RNF2). This promotes further chromatin compaction and transcriptional repression [139].

Other histone methylation marks contribute to chromatin compaction and repression, including H3 Lys9 trimethylation (H3K9me3). In a mechanism reminiscent of that for H3K27me3 establishment, H3K9 is methylated through the combined actions of the HMT SUV39H1/2 and a reader-writer coupled HP1 CBX protein. SUV39H1/2 is recruited to H3K9me1 to deposit H3K9me3 and recruit other repressive proteins [141]. Both H3K9me3 and H3K27me3 are crucial for maintaining heterochromatic domains which may be clonally inherited.

In marking active transcription, H3K36me2/3 significantly impairs H3K27 di- and trimethylation by PRC2 in a cis-nucleosomal fashion, indicating that H3K36me2/3 inhibits the spread of heterochromatic markers [142]. H3K36me3 is deposited along actively transcribed regions through recruitment of SETD2 to RNA polymerase (pol) II. This serves to induce chromatin compaction as pol II passes through the open reading frame, through a mechanism not yet fully elucidated in mammals. Ultimately, H3K36me3-induced compaction prevents transcription from initiating on intragenic cryptic promoters, disallowing incomplete mRNA synthesis (as reviewed in [143]).

Another prominent category is H3 lysine 4 methylation (H3K4me), which differentially marks active regions based on methylation signature. H3K4 methylation is carried out by mixed-lineage leukemia (MLL/KMT2A) SET-domain containing proteins. Generally, H3K4me1 marks regulatory enhancer elements, whereas H3K4me2/3 are associated with actively transcribed genes, with H3K4me3 localized near the transcription start site and H3K4me2 deeper in the gene body [144]. Methylated H3K4 can promote transcription by recruiting elongation factors. For example, H3K4me2/3 recruits the human CHD1 chromatin remodeling protein, a key transcriptional co-activator which can evict nucleosomes ahead of RNA pol II [145]. A caveat to enhancer delineation with H3K4me1 is that it is not always indicative of an active enhancer. H3K4me1 can prime enhancers for activation, but functional enhancers are specifically marked by acetylation of H3 Lys27 (H3K27ac) (reviewed in [146]).

There are many examples of epigenetic dysregulation even in cancers with known genetic components. In one study of histone state switching in leukemia, MLL1 was found to promote and maintain stem cell markers in intestinal cancers through Wnt signaling and conversion from H3K27me3 to H3K4me3 at their promoters [147]. Epigenetic mechanisms involving PRC2, Wnt, HDACs, and other factors also feature in the repression of epithelial-specific gene expression and epithelial-to-mesenchymal transition (EMT) characteristic of many metastatic cancers, and regulate stability of cellular identity and resistance to dedifferentiation [148].

#### Histone acetylation at Lys27

Unlike histone methylation, histone acetylation directly affects histone-DNA interactions. The transfer of an acetyl group to the $\epsilon$-amino group of lysine converts the normally positively charged ammonium group to an inert amide. This masks the positive charge of lysine, reducing attractive interactions between the histone tail and DNA. Histone acetylation is carried out by type-A histone acetyltransferases (HATs) in the nucleus, split into three main families: the GCN5-related N-acetyltransferases (GNATs, including KAT2A and KAT2B), MYST proteins, and the p300/cyclic AMP response element binding protein (CREB) binding protein (CBP) family. HATs commonly have a central conserved acetyl CoA (acetyl group donor) binding unit with variable flanking N- and C-termini that may confer substrate specificity [138].

H3K27ac is considered a strong readout for active enhancer elements due to correlation of H3K27ac occupancy with H3K4me1 occupancy at intergenic/intronic sites and expression of genes proximal to H3K27ac, as well as anti-correlation with repressive marks like H3K27me3. Additionally, the HAT p300 delineates both poised (H3K27me3-repressed) and active enhancers [149]. P300 is a key enzyme involved in the acetylation of H3K27, implying that enhancer-associated H3K27ac may be regulated two-fold by controlling the recruitment and catalytic activity of HATs [146]. Recent studies continue to unravel the mechanisms of H3K27 acetylation at enhancer elements. The pioneering transcription factor c-Myb has been shown to recruit p300 to acetylate H3K27 at enhancer elements; mutation of its DNA-binding domain resulted in decreased gene expression and similar H3K27ac signatures to cell lines without c-Myb transfection in vitro [150]. Pioneering transcription factors are those able to bind nucleosomal DNA otherwise inaccessible to DNA-binding proteins. Such factors allow loading of other transcription factors or chromatin remodeling complexes for de novo activation of regulatory elements, and are frequently involved in cell fate reprogramming [146]. Further regulation of p300 may involve its acetyllysine-recognizing bromodomain. Raisner et al. demonstrated that small molecule inhibition of the p300/CBP bromodomain reduces H3K27ac signatures at enhancer elements without affecting p300 localization, H3K18ac, or H3K4me levels at enhancers. The loss of H3K27ac was concomitant with a decrease in gene expression at proximal genes, implicating H3K27ac as a causal agent for transcriptional activation [151]. An attractive regulatory circuit to explain H3K27 acetylation then relies on pioneering factors to recruit HATs to closed enhancers, possibly also allosterically activating their catalytic domain to facilitate enhancer activation. However, this may oversimplify the full picture, and additional layers of H3K27ac regulation continue to be discovered.

Various transcriptional activators bind in patterns overlapping enhancers. For example, the pause release factor BRD4, which indirectly antagonizes negative elongation factor to promote productive elongation, can associate with enhancer elements. Impairment of p300 catalytic activity diminishes this association [152]. Remarkably, assembly of the pre-initiation complex and transcriptional activity at enhancers has been documented, with the resultant RNA species being coined “enhancer RNAs” (eRNA). Production of eRNA is correlated with proximal mRNA production, and mounting evidence implicates eRNA as a bona fide functional effector of gene expression (reviewed in [146]). In one recent example in murine T-ALL, eRNA ARIEL was shown to trigger Myc and other oncogenic signaling through activation of the ARID5B enhancer [153].

Finally, dense occupancy of H3K27ac is a key feature of super-enhancers: enhancer elements that are larger than typical enhancers and ubiquitously bound by Mediator and cell-type specific master transcription factors that control cell fate. Early studies established that common features of active enhancers (e.g., H3K27ac, H3K4me1, and DNase hypersensitivity) are amplified at these domains, which are also leveraged in oncogenesis and other disease processes [154]. In one recent report, super-enhancers were H3K27ac-bound in cancer type-specific patterns [155], as well as prospective upstream regulators of known oncogenes. A super enhancer also regulates expression of PD-L1 and PD-L2 [156], and thus may feature in immune evasion and subclone-specific resistance to immunotherapy. Overall, H3K27ac is intimately and intricately associated with gene expression programs through enhancer activity.

#### DNA methylation

DNA methylation, or the addition of methyl groups to create 5-methylcytosine in cytosine-guanine dinucleotides in DNA, has a long-recognized role in cancer biology and provides an important readout of epigenetic state [157–159]. Promoter DNA methylation represses gene transcription, producing stable silencing in the absence of inactivating mutations. DNA methylation directs genomic imprinting, X-inactivation, inhibition of transposable elements, and certain forms of chromatin compaction, with a general role in long-term silencing of heterochromatic domains.

**Fig. 5:**
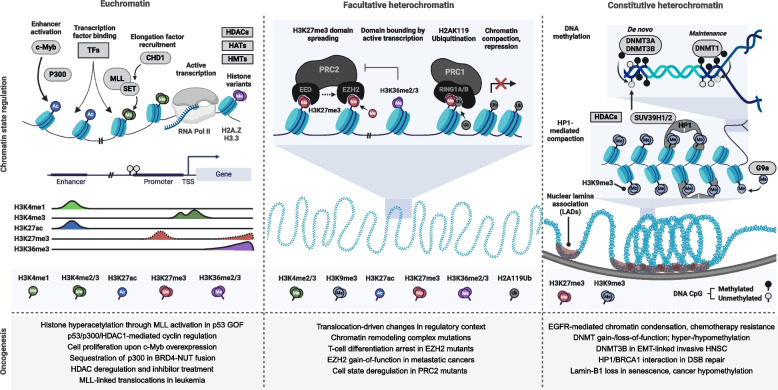
Chromatin state regulation and misregulation in cancer. Nuclear chromatin can be broadly clustered into domains of euchromatic (left) and heterochromatic character, divided into facultative (middle) and constitutive (right) classes. (Left) Euchromatic regions contain transcriptionally competent genes with high levels of enhancer H3K4me1 and H3K27ac, high H3K4me3 bounding transcription start sites (TSS), and repressive promoter H3K27me3 levels that depend on local regulation. H3K36me3 marks recently transcribed genes to suppress intragenic transcription. (Middle) Facultative heterochromatin is characterized by broader H3K27me3 domains established by PRC2, bounded by H3K36me2/3, and H2AK119 ubiquitination by H3K27me3-directed PRC1. (Right) Constitutive heterochromatin features high H3K9me3 and consequent HP1 binding adjacent nucleosomes to direct chromatin compaction, along with domains tethered to the nuclear lamina (LADs). DNA methyltransferases stabilize silencing and compaction of facultative and constitutive heterochromatic domains. (Bottom) Loss of regulated transitions between these states are common in oncogenic transformation, with representative examples shown

The signs of aberrant DNA methylation are extensive, found in dozens of cancer types and hundreds of marker genes. Sequencing of somatic mutations, germline polymorphisms, and methylation panels have been applied to model risk in colorectal, lung, prostate, and other cancers [160–164]. These risks are commonly increased by hypermethylation-mediated silencing of tumor suppressors. Conversely, loss of maintenance or de novo DNA methyltransferase activity from Dnmt3 or Dnmt1 (Fig. 5) can lead to a failure to repress growth factors, proliferation signals, and other oncogenes [165, 166]. Methylation state can also help predict cases of targeted resistance to chemo- or immunotherapy and contribute to immune evasion [167, 168]. While most gene-regulatory DNA methylation occurs in CpG islands in promoters, other regulatory connections extend to and from noncoding RNA species [169].

As with other layers of epigenetic regulation, changes in DNA methylation often occur in concert (up- or downstream) with those in H3K27me3, HP1, nuclear lamin binding, or other chromatin elements. Thus, while some DNA methylation changes indicate specific gains or losses of local regulation, others reflect broader stabilization of a repressive chromatin state. Comprehensive overviews of the roles of DNA methylation in oncogenesis [155], metastasis [170], familial cancer risk [171], and diagnosis [172] provide more thorough coverage for this important topic.

#### Chromatin state regulation

Chromatin can be broadly categorized into spatially segregated euchromatic or heterochromatic domains based on the additive effects of histone modifications and nucleosome compaction. The former consists of more accessible, early replicating, and transcriptionally competent regions, whereas the latter is transcriptionally repressed, generally localized to the nuclear periphery, and replicates late in S phase [173] (Fig. 5).

Euchromatin is gene-rich and delineated by histone modifications associated with active transcription such as H3K27ac, H3K4me, and H3K36me. Euchromatic regions are sites for high transcriptional activity and are preferentially found within the nuclear interior [173]. Euchromatic domains are also demarcated by H2A.Z and H3.3 variants that support active gene expression. Nucleosomes containing H2A.Z are generally less stable and more mobile along chromatin than their canonical counterparts, with H2A.Z/H3.3 double variants susceptible to nucleosome eviction. Consequently, H2A.Z is frequently found in the immediate downstream nucleosome of a transcriptional start site and at enhancer regions, where it may facilitate the depletion of nucleosomes to make these regions more accessible [174]. On a similar note, chromatin remodeling complexes play a key role in mediating DNA accessibility within euchromatin. For example, the SWI/SNF family of remodeling complexes are largely responsible for catalyzing nucleosome sliding along and eviction from chromatin, and are recruited to acetylated histones commonly found in euchromatin [175].

In contrast, heterochromatin is repressive, displays low accessibility, and is typically marked by the repressive complement of histone modifications. Heterochromatin can be further divided into two subtypes: facultative and constitutive. Facultative heterochromatin tends to occur across regions containing developmental genes and can vary in response to growth factors and environmental stimuli. Constitutive heterochromatin is generally stable, and occurs around repetitive and pericentromeric regions [176]. Specifically marked by H3K9me3, constitutive heterochromatin spreads through reader-writer coupling of SUV39H1/2 and CBX proteins, including HP1. By contrast, facultative heterochromatin is marked by H3K27me3 deposited by PRC2, spreading through the combined actions of EED methyl reading and EZH2. PRC2 can also be recruited to CpG islands in a H3K27me3-independent fashion through the SUZ12 subunit, suggesting a role for SUZ12 and other PRC2 accessory factors in de novo formation of facultative heterochromatin [177].

Importantly, domains of chromatin state can be clonally inherited. The parental H3-H4 tetramers of the histone core are divided between sister chromatids during replication, resulting in interspersed nucleosomes with parental histone marks occupying daughter chromatin [178, 179]. Repressive marks can serve as platforms for SUV39H1/2 or PRC2 to bind and nucleate repressive marks on the neighboring nucleosomes assembled from free histones, re-establishing the heterochromatic domain [180]. Accordingly, Dnmt1 binds hemimethylated CpG sites to reconstruct DNA methylation on the newly synthesized strand [181]. Finally, a dynamic balance is struck at the borders of heterochromatin and euchromatin to regulate these domains. Many distinct mechanisms can contribute to this equilibrium: histone modifying enzymes can restrain the spread of heterochromatin, DNA elements can disrupt heterochromatin writers, or nucleosome-free regions can isolate reader-writer coupled heterochromatic enzymes from spreading into euchromatic regions [176]. The regulation of chromatin state is fundamental in cellular homeostasis and in driving cell lineage-specific expression programs.

In addition to individual loci, entire chromatin domains can be deregulated or found in cancer-specific states (Fig. 5). This can occur through domain-wide silencing of tumor suppressors or activation of oncogenes, as in EZH2 gain-of-function mutations [182] and p300 sequestration and differentiation arrest through BRD4-NUT fusion [183, 184]. Translocation-driven alterations in three-dimensional positioning can also bring domains into improper regulatory contexts. Similar effects have been observed in leukemias, in which replication timing profiles demonstrated consistent changes in leukemic cell lines and patient samples that followed normal boundaries of changes in development [185]. Some forms of treatment resistance are also affected by chromatin state. KRAS mutant lung cancers appear to acquire radiation therapy resistance through EGFR-mediated chromatin condensation, thereby blocking induction of DSBs [186]. More generally, domain-scale alterations in chromatin state offer mechanistic insights into the etiology of cancers with few or no known genetic origins.

### The 3D genome

Beyond the partitioning of euchromatin and heterochromatin, spatial organization is a key determinant of gene expression. The higher-order folding of chromatin can map regulatory elements to genes under their control, such as in promoter-enhancer loops, insulate such interactions to alter gene expression patterns, or establish and maintain chromatin states. Here, we discuss current efforts to unravel genome topology and its consequences, as well as technology used to interrogate the three-dimensional genome.

#### Genome compartmentalization

Our current understanding of genome structural regulation has been greatly aided by the development of all-vs-all, quantitative measures of genomic contacts. First reported in 2009 by Lieberman-Aiden et al., Hi-C is a genome-wide application of chromatin conformation capture technology originally developed by Dekker’s group [187]. Hi-C utilizes the fact that interacting genomic regions must be spatially adjoined regardless of their relative position in the linear genome to capture and sequence these pairs. Following a size selection step and PCR amplification, reads of interacting loci can be sequenced and mapped back to the genome, creating a map of average interaction behavior across a population of cells [187]. To visualize and perform computation on the results of Hi-C alignment, the genome is first tiled into bins of fixed width, and the number of contacts between each bin are counted. Scrutiny of such contact maps has revealed new dimensions of genome organization and regulation.

Examination of 1 Mb resolution Hi-C maps of lymphoblastoid cell lines revealed that intrachromosomal loci were naturally divisible into two self-interacting groups, such that within-group contact profiles were correlated and between-group contact profiles were anti-correlated. Arbitrarily assigned labels of group A or B, these compartments were found to be strongly associated with chromatin state, with A compartment regions associated with activating histone marks, DNase hypersensitivity, and gene expression, and the B compartment with repressive histone marks and DNase insensitivity. The consistency of this pattern for all nuclear chromosomes led to the conclusion that the nucleus is largely organized into two major spatial categories: the active and euchromatic A compartment, and the repressive and heterochromatic B compartment [187]. Loci tend to interact with loci residing in the same compartment moreso than the opposite compartment, forming “megadomains” [187, 188]. Follow-up work defined subcompartments A1, A2, B1, B2, and B3, in which A1 replicates earlier in S-phase than A2, B1 is enriched in H3K27me3 marks characteristic of facultative heterochromatin, and B2 consists largely of pericentromeric (constitutive) heterochromatin [188].

A central insight from Hi-C experiments has been the notion that chromosomes can be subdivided in the context of regional contacts, with domains on the order of several hundred kilobases to megabases exhibiting enriched intra-domain and depleted inter-domain contacts. We next consider the role of these domains in regulating gene expression in the context of cancer.

#### Chromosome organization and topologically associating domains

**Fig. 6:**
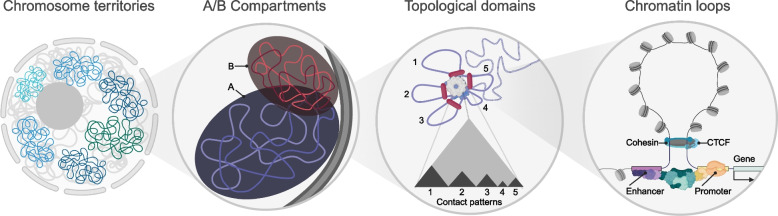
Scales of genome structural regulation. Chromosome territories divide the nucleus into a top level regional organization. Beyond this, partitions emerge of largely self-interacting compartments of euchromatic (compartment A) or heterochromatic (compartment B) character, associated with domain-level repression or activation. Topological domains represent individual regions of enriched interactions. At the smallest scales, misregulation can occur through loss or gain of architectural protein interactions and enhancer-promoter contacts

Perhaps the largest unit of the nuclear hierarchy are chromosome territories (CTs), which describe longstanding observations that eukaryotic chromosomes occupy largely distinct and discrete spaces within the nucleus. While it is yet to be fully elucidated how these territories arise, some lines of evidence suggest that CTs may adopt a non-random radial distribution from the nuclear center and adjoining sites [189], and are specific to individual cell states (Fig. 6).

Contact profile analysis of many lineages has further revealed folding properties of chromosomes within their respective territories. Dixon et al. discovered that chromosomes can be decomposed into a series of domains, within which contacts are enriched, separated by boundary sites which insulate contacts with external loci. They named these domains “topologically associating domains” (TADs), which were on the order of hundreds of kilobases to megabases [190]. Domain boundaries were defined as sites in which the directionality index, quantifying the frequency with which a locus interacts with upstream or downstream regions, abruptly changes direction, indicating a shift towards distinct contact profiles of linearly adjacent loci. Dixon and colleagues noted that CTCF binding was enriched at TAD boundaries, and high resolution Hi-C studies confirmed that chromatin loops are preferentially anchored by CTCF [188]. TADs tend to be conserved across cell types, implicating TADs as a fundamental feature of eukaryotic chromosome folding [190].

Unsurprisingly, TADs play critical roles in the regulation of developmental genes, in part by regulating other features of the epigenome. Narendra et al. demonstrated that deletion of a CTCF insulator site that separates the HoxA cluster into an active and repressive TAD in ESC-derived motor neurons results in spreading of active histone marks to the repressed domain, inducing gene expression [191]. By contrast, expression of Sonic hedgehog (Shh), often co-opted in cancer and a factor in treatment resistance, appears unaffected by local TAD disruption but dependent on long-range enhancer contacts through cohesin [192, 193]. Together, results suggest that TADs act as effectors to partition active and repressed chromatin and link enhancers and gene targets, but probabilistically and in concert with other layers of regulation.

In cancers, A/B compartment character and TAD insulation have emerging roles in misregulation. Acute myeloid leukemias, often karyotypically normal but with differentiation arrest, have been recently found to harbor characteristic alteration in TADs that enhance or silence genes required for leukemogenesis [194]. Mutations in the STAG2 subunit of cohesin, which surrounds endpoints of chromatin loops and mediates promoter-enhancer contacts (Fig. 6), result in overexpression of the HOXA cluster and downregulation of MAPK pathway members [195]. TAD structure and cohesin function are implicated in the gain of loop boundaries associated with HOTTIP lncRNA, which activates Wnt signaling targets and HOXA genes in maintaining leukemic progenitor phenotypes [196, 197].

As a factor in cell differentiation, genome organization is in turn closely connected to immune function. Loop extrusion at the Igh locus is the architectural basis for V(D)J recombination, providing the substrate for antigen-specific antibody production in the adaptive immune response [198]. The cohesin release factor WAPL mediates this process in a manner dependent on Pax5 [199], which is itself disrupted in a wide array of leukemic subtypes [200]. This may have broader implications for treatment. For instance, the competence of PD-1 checkpoint blockade treatments appears to be mediated through local chromatin state and insulation of the domain containing CD274 and CD273 [156], encoding the programmed death ligands PD-L1 and PD-L2 often involved in immune evasion.

## Metastasis

### Models of metastasis

Current concepts of metastasis largely focus on the development of invasive subclones within a tumor in the context of clonal evolution. Selection pressures for aggressive subclones arise both within the tumor microenvironment and from external factors that otherwise prevent establishment at distant organs. Such factors include cell-cell adhesions, the extracellular matrix, oncogene-induced stress, and rate-limiting steps over the course of metastatic progression such as surviving blood vessel intravasation and extravasation [201]. The innate genomic instability of cancer and resulting tumor heterogeneity serve as the building blocks on which these pressures exert their effects.

The point at which cells with metastatic potential arise is currently debated. One theory posits a linear model, in which the development of the primary tumor predates the evolution of a metastatic subpopulation, while the alternative parallel progression model suggests that subpopulations with a propensity to disseminate evolve in tandem with primary tumor shortly after cancer initiation by a founder cell. Further, evidence suggests a possible dichotomous mode of metastasis spread, in which either a single metastatic subpopulation creates secondary sites at multiple tissues (monophyletic seeding), or that multiple metastatic subpopulations evolve within a primary tumor and each establish at new organs in the body (polyphyletic seeding) [202]. The trajectory of metastasis evolution has broad implications for colonization targets and genomic divergence between primary and secondary sites, key considerations for anti-cancer therapies.

The many bottlenecks that must be overcome by cancer metastases elicits a natural question: what cellular or biochemical processes enable these tumor cells to adopt aggressive and invasive phenotypes?

### Epithelial-to-mesenchymal transition

**Fig. 7:**
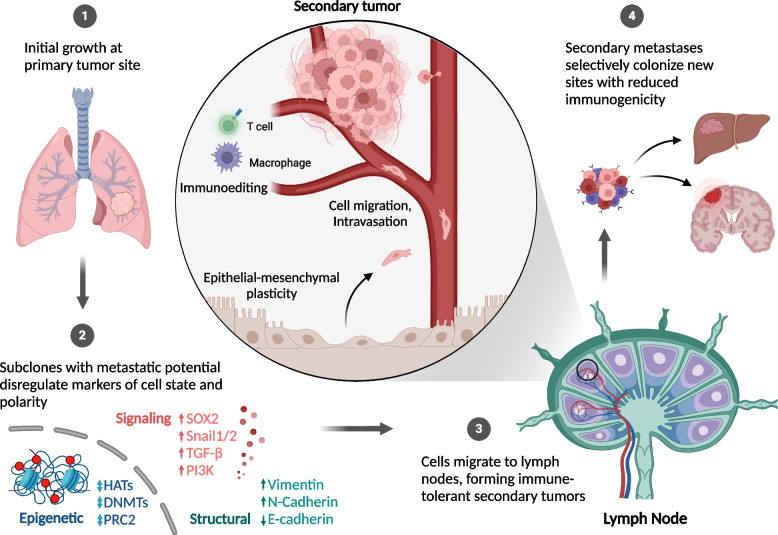
Cell state deregulation, migration, and metastasis. Pre-metastatic cells lose epithelial features and silence expression of epithelial biomarkers such as E-cadherin in favor of a more aggressive mesenchymal phenotype, characterized by expression of vimentin and N-cadherin and often accompanied by stem cell markers. Loss of cellular identity and adhesion promotes migration, lymph node engraftment, and development of immune-tolerant secondary tumors

The epithelial-to-mesenchymal transition (EMT) is a vital process in embryonic development and carcinoma progression and metastasis (Fig. 7). EMT is broadly characterized by the loss of cellular adhesion and apical-basal polarity that influences cell junction organization, hallmarks of epithelial tissue. Cells undergoing EMT preferentially co-opt a more motile and aggressive mesenchymal phenotype, which allows them to detach from the basement membrane and invade nearby biological structures [203].

EMT is induced through a core set of transcription factors that serve to repress genes that participate in the epithelial phenotype. The zinc-finger transcription factors Snail1 and Snail2 bind regulatory E-box motifs in the E-cadherin promoter and repress its transcription. In particular, nuclear levels of Snail1 can be potentiated through canonical Wnt signaling in breast cancer cells, promoting aggressive phenotypes [204]. The zinc-finger E-box binding homeobox proteins 1 and 2 (ZEB1/2) have also been implicated in EMT; in vitro prostate cancer models revealed that ZEB1 binds to the E-cadherin promoter and recruits the histone deacetylase Sirtuin-1, resulting in histone deacetylation and repression of E-cadherin [205]. The transcription factor Twist1 can indirectly contribute to the EMT cascade by transactivating Snail2, resulting in Snail2-mediated repression of E-cadherin. Deletion of Snail2 blocks the activation of mesenchymal biomarkers, inhibiting EMT [206]. These EMT-inducing genes are associated with cancer metastasis. For example, evidence suggests that ZEB1 activates the H3K4me3 writer SETD1B in colorectal carcinoma, leading to a positive feedback loop of transcriptional potentiation of ZEB1 and thus EMT. Concordantly, higher levels of ZEB1/SETD1B expression were associated with spindle-shaped mesenchymal cells and poorer patient prognosis [207].

In concert with these EMT transcription factors, signaling through the transforming growth factor $\beta$ (TGF-$\beta$) pathway can also trigger the transition to a more migratory phenotype. The TGF-$\beta$ superfamily consists of many subfamilies of related proteins, including the canonical TGF-$\beta$ subfamily, the bone morphogenetic protein family, activins, and nodals. Canonical TGF-$\beta$ proteins exert their effects through a heterotetrameric receptor complex consisting of two subunits each of TGF-$\beta$ receptor type 2 (TGFBR2/TGFRII) and TGF-$\beta$ receptor type 1 (TGFBR1/TGFRI). Upon TGF-$\beta$ binding to the extracellular ligand domain, the receptor kinase TGFBR2 phosphorylates and activates TGFBR1, which goes on to phosphorylate its targets, the receptor-activated SMAD2 and SMAD3 [208]. These SMADs act as key mediators in TGF-$\beta$-induced EMT, as in vivo mice models knocked out for renal expression of SMAD3 showed decreased levels of Snail and mesenchymal marker $\alpha$-smooth muscle actin compared to wild-type following induction of renal fibrosis [209]. EMT is a potent tool for molding migratory, invasive subpopulations within a primary tumor. However, it is certainly not the only driving force behind cancer metastasis.

### Metastatic signatures in chromatin state

Rather than through isolated mechanisms, metastatic changes in cell state occur through coordinated transformations in epigenetic, signaling, structural, and transcriptional elements. As one readout of such states, chromatin accessibility changes have also emerged as a distinguishing feature of metastasis. In addition to direct effects on oncogenic and metastatic drivers, the accessibility landscape directs access to DNA binding proteins and chromatin regulators, and these can be down- or upstream of genetic lesions. In models of liver metastasis from small cell lung carcinoma, copy amplification of NFIB (Nuclear factor I B) produced genome-wide accessibility increases [210]. Sites of differential accessibility were enriched in Nfib binding sites and occupancy, suggesting a model in which Nfib binding competitively depletes nucleosomes to increase expression of target genes. Surprisingly, regulation appeared to be direct, with Nfib knockdown reversing 82% of differentially accessible regions and producing several-fold reduction in liver metastases [210]. Related Nfib, Myb, and MAPK/MEK signaling defects commonly arise in adenoid carcinomas with neural invasion, with over 60% of cases exhibiting NFIB::MYB or NFIB::MYBL1 fusion [211, 212].

Such epigenetic and transcriptional effects in metastasis are often interlinked. SETD2 is an H3K36 trimethylase mutated in roughly 20% of in clear cell renal carcinoma (ccRCC) cases and nearly half of metastases. Loss of SETD2 function leads to global H3K36me3 depletion, increases in chromatin accessibility, and higher rates of metastasis to lung, liver, and brain, with recovery of normal accessibility and metastasis patterns upon SETD2 or H3K36me3 rescue [213]. Many of these effects would be missed using whole-exome mRNA sequencing, as the majority were observed across introns and intergenic regions, and through increased H3K27ac and H3K4me1 occupancy representing enhancer activation distal to target genes, with activation signatures of MYC, STAT, and loss of PTEN. In this case, a genetic mutation produces an epigenetic impact on the regulation of well-known oncogenes.

Metastasis-linked structural events have been similarly traced across metastatic ccRCC clones by the TRACERx Renal Consortium and others [214]. In ccRCC the genetic elements of primary disease are often well-defined: von Hippel-Lindau tumor suppressor (VHL) is disrupted in over half of ccRCC cases, and lower metastatic potential is associated with a higher degree of genetic homogeneity in primary tumors affected by fewer somatic copy number alterations. Primary tumors cluster into two populations: one of homogeneous tumors showing rapid progression to multiple secondary sites, and a second of highly heterogenous tumors with more gradual progression to a single secondary site [214]. Thus, modes of metastasis can be driven by specific tumor etiology, and broad heterogeneity at the primary tumor can serve as the primary substrate for selection of metastasis-competent clones.

More recently, advances in single-cell methods have been leveraged to track the etiology and evolution of metastatic clones, connecting mechanisms in EMT to paired DNA accessibility and expression changes. Using samples of 11 tumor types from the NCI Human Tumor Atlas Network (HTAN) and Clinical Proteomic Tumor Analysis Consortium (CPTAC), Terekhanova and colleagues applied single-nucleus ATAC-seq to identify regions of differential accessibility, finding both site-specific and pan-cancer signatures [215]. These aligned with mechanistic and possible treatment targets, including EMT regulators TWIST1, PBX1, GATA6, and ELF3. Reduced accessibility in GRHL1 was observed in metastatic cancers, which may suppress epithelial cell adhesion and promote migratory phenotypes, while accessibility changes also overlapped mutation hotspots for TERT and promoters of several FOX transcription factors. Effects were further coupled to prognosis, as event-free survival was sharply associated with PITX3 and KLF6 regulation [215]. Along with consistent results on cancer-type specificity of metastatic drivers across 23 cancer types [170], similar recent work has strengthened connections between layers of epigenetic regulation and the misexpression of cancer driver genes.

### The search for other metastatic signatures

Cancers are largely the result of genetic lesions sustained in strategic regions of the genome that result in misregulation of proliferation and cell homeostasis. Given the mutational signatures known to confer oncogenic potential in somatic cells, one may question whether there is a “metastasis signature” of mutational injury that resolves secondary tumors from their primary counterparts. Currently, no such general mutation profile exists. Present studies suggest that the answer may be tissue type and tumor dependent. A cohort study of 41 Sardinian cases of cutaneous malignant melanoma found that metastatic samples showed mutational concordance with primary tumors for known cancer driver genes [216]. Similar analysis of driver gene mutations in pancreatic ductal adenocarcinoma also revealed concordance between metastases and primary tumors [217]. Studies in other cancers demonstrate that driver genes are frequently mutated early in tumorigenesis and are thus clonally inherited by all descendent lineages, including metastases, such that necessary pro-proliferative and pro-migratory lesions may already be sustained in the primary tumor (reviewed in [202]). However, this may not be a universal phenomenon—these results have been challenged in metastatic breast cancer, where additional mutations in metastases beyond those inherited from their primary tumors resulted in misregulation of key pathways including PI3K, HIF-1, and VEGF signaling [218]. Altered transcriptional regulation likely contributes to metastatic potential in many tumor subclones. In multiple cancer types, epigenetic reprogramming through large scale differences in chromatin accessibility [210], differential enhancer activation [219], and the activation of oncogene-associated super-enhancers [220] all contribute to cancer metastasis. In conjunction with the results of mutational concordance between metastases and primary tumors, this suggests that genome re-organization may be a necessary process in the evolution of metastasis.

### Conclusions and future directions

The rising health and healthcare burden of cancer and the high toll of metastatic cancers create a correspondingly powerful incentive to understand their molecular pathophysiology. However, identifying driver events that specifically resolve metastasis from primary tumor development remains a major challenge. Many common hallmarks of oncogenesis support metastatic spread through their inherent nature of promoting aggressive proliferation and growth. A meta-analysis by Lukashchuk and colleagues found that advanced, metastatic prostate cancers show significantly increased rates of mutation in homologous recombination repair enzymes, including BRCA1/2, than primary prostate cancers [221]. Similarly, increased frequency of chromosomal mis-segregation (and thus increased chromosomal instability) in diffuse large B-cell lymphoma is correlated with bone marrow involvement and poor overall survival [222]. Genomic and chromosomal instability naturally create stochastic perturbations of genome state that can serve to increase tumor heterogeneity, creating a broad molecular landscape within a tumor that may give rise to disseminating clones [223–225]. Yet, identifying the point at which such aberrations occur in clonal evolution and whether they are causal events or manifestation of larger genomic reprogramming in metastasis remains to be decisively determined.

Epigenetic reprogramming events can also be shared between primary and metastatic tumors. EZH2 over-expression triggers cancerous transformation of many tissue types through H3K27me3 modulation, as part of PRC2-dependent and independent mechanisms [226, 227]. Global changes in H3K27me3 occupancy by way of EZH2 active site mutations promote B-lymphoid neoplasms [228, 229]. In addition to supporting primary neoplastic growth, EZH2 has been well-implicated in metastasis. Tong and colleagues discovered EZH2 can participate in epigenetic suppression of E-cadherin in nasopharyngeal carcinoma through interactions with histone deacetylases 1 and 2, promoting invasion [230]. Such findings reinforce the notion that many driver events for tumorigenesis also impose selective advantages for propagation, but raise questions regarding the metastatic potential of early clones. Extracellular factors also influence the invasive capabilities of tumor subclones within a population. The tumor microenvironment plays integral roles in cancer and metastasis through many mechanisms: immunosuppression through secretion of anti-inflammatory cytokines (e.g., IL-10) by tumor-associated macrophages [231, 232], promoting migratory phenotypes through activation of pathways such as Wnt and EGFR [233, 234], induction of hypoxia and secretion of pro-angiogenic factors [235, 236], and promoting chemoresistance [237]. This complex interaction of genomic, epigenomic, and environmental insults is a major factor in promoting dissemination.

Ultimately, significant work remains to understand the evolution of the genome and 3D epigenome in metastasis. This will require direct comparisons between tumor source and destination tissues aided by advanced -omics approaches to understand transitions in the state of individual cells. Advances in single-cell sequencing and lineage tracing experiments provide promising avenues to better understand metastasis as dynamic processes in response to selective pressures to retain a fitness advantage in different tissue environments [238, 239]. Although many of these mechanisms are likely tumor type-specific, others may be shared, and each may serve to explain longstanding observations of heritable cancers with no known genetic component.

## Data Availability

No datasets were generated or analysed during the current study.

## Abbreviations

APC: Adenomatous polyposis coli
APC/C: Anaphase-promoting complex/cyclosome
ATM: Ataxia-telangiectasia mutated
ATR: Ataxia telangiectasia and Rad3-related protein
BRCA1: Breast cancer gene 1
CDK: Cyclin-dependent kinase
CRC: Colorectal cancer
CHEK2: Checkpoint kinase 2
DALY: Disability-adjusted life year
DSB: Double-strand break
E2F: E2 transcription factor family
EGF: Epidermal growth factor
EMT: Epithelial-to-mesenchymal transition
ERK: Extracellular signal regulated kinase
FGF4: Fibroblast growth factor 4
GEF: Guanine nucleotide exchange factor
GSK3: Glycogen synthase kinase 3
HDAC: Histone deacetylase
HER2: Human epidermal growth factor receptor 2
KMT2A: Lysine methyltransferase 2A
LEF: Lymphoid enhancer factor
LRP5: Low-Density lipoprotein receptor-related 5
MAPK: Mitogen-activated protein kinase
MCM: Minichromosome maintenance
MEF: Myocyte enhancer factor
MLL: Mixed-lineage leukemia
MRN: MRE11-RAD50-NBS1 complex
mTOR: Mammalian target of rapamycin
NF-$\kappa$B: Nuclear factor kappa B
NHEJ: Non-homologous end joining
PcG: Polycomb group
PI3K: Phosphoinositide 3-kinase
POLD1: Polymerase delta 1
POLE: Polymerase epsilon
pre-RC: Pre-replication complex
Rb: Retinoblastoma protein
Shh: Sonic hedgehog
SOX2: SRY-related HMG box 2
STAT3: Signal transducer and activator of transcription 3
TAD: Topologically associating domains
TCF: T cell factor
TERT: Telomerase reverse transcriptase
TGF-$\beta$: Transforming growth factor $\beta$
TGFBR: Transforming growth factor $\beta$ receptor
TP53: Tumor protein p53
TP53BP1: Tumor protein p53 binding protein 1
ZEB1: Zinc-finger E-box binding homeobox protein 1
